# Politeness in Human–Robot Interaction: A Multi-Experiment Study with Non-Humanoid Robots

**DOI:** 10.1007/s12369-022-00911-z

**Published:** 2022-08-18

**Authors:** Shikhar Kumar, Eliran Itzhak, Yael Edan, Galit Nimrod, Vardit Sarne-Fleischmann, Noam Tractinsky

**Affiliations:** 1 Department of Industrial Engineering and Management, Ben-Gurion University of Negev, Be’er Sheva, Israel; 2 Department of Communication Studies, Ben-Gurion University of Negev, Be’er Sheva, Israel; 3 Department of Information Systems Engineering, Ben-Gurion University of Negev, Be’er Sheva, Israel; 4 The Interdisciplinary Department of Social Sciences, Bar Ilan University, Ramat Gan, Israel

**Keywords:** Politeness, Human Robot interaction, Older adults, Social assistive robot, Assistive robot

## Abstract

We studied politeness in human–robot interaction based on Lakoff’s politeness theory. In a series of eight studies, we manipulated three different levels of politeness of non-humanoid robots and evaluated their effects. A table-setting task was developed for two different types of robots (a robotic manipulator and a mobile robot). The studies included two different populations (old and young adults) and were conducted in two conditions (video and live). Results revealed that polite robot behavior positively affected users' perceptions of the interaction with the robots and that participants were able to differentiate between the designed politeness levels. Participants reported higher levels of enjoyment, satisfaction, and trust when they interacted with the politest behavior of the robot. A smaller number of young adults trusted the politest behavior of the robot compared to old adults. Enjoyment and trust of the interaction with the robot were higher when study participants were subjected to the live condition compared to video and participants were more satisfied when they interacted with a mobile robot compared to a manipulator.

## Introduction

Acceptance of any technology in a society is highly dependent on functional and social aspects [1]. Accordingly, the social behavior of robots is considered influential on humans’ willingness to interact with them [2]. Here we consider one aspect of such behavior—politeness. Politeness has an important role in human social behavior, helping humans increase interaction amongst themselves and avoid conflict. According to Lakoff [3], politeness is “*a system of interpersonal relations designed to facilitate interaction by minimizing the potential for conflict and confrontation inherent in all human interchange*”. The leap from politeness as a key construct in human–human behavior to its role in human-technology communication may not seem straightforward. Yet, in the field of human–computer interaction (HCI) media equation theory suggests that humans treat machines similar to other humans [4]. Computer etiquette was suggested as an important factor while interacting with humans [5]. Developing etiquettes could influence humans’ trust in automation as it would help them understand the way automation works [6]. Trust is the ability of the trustee to perform a significant action based on the expectation of the trustor which the trustor could rely upon [7, 8]. Accordingly, it could help bridge the gap between humans’ expectations and the agent’s functionality [6]. Further empirical research has shown that people tend to be polite towards the computer after a conversation has been initiated [9]. Consequently, research has also argued for the study of politeness in HRI [10–12]. Subsequent empirical research on the topic has focused mainly on tasks with humanoid robots, e.g. [13–18]. No study, thus far, explored politeness in non-humanoid robots according to our knowledge.

This study focuses on studying the effects of social robots’ politeness in human–robot collaborative tasks. For this purpose, we have adapted a sociolinguistic approach to politeness in general [3, 19] and particularly in the field of HCI [20]. We elaborate on this approach in Sect. 2.1. We have developed three different levels of robot politeness based on the politeness rules outlined by [3]. The politeness rules have been incorporated in a human–robot task with different non-humanoid robot types (mobile and manipulator robots). Further, we evaluated the influence of politeness in several user studies both by a video and live experiment with both old adults and young adults. In a previous experiment, we performed a preliminary investigation with old adults and young adults with a manipulator robot [21]. In the current paper, we compare these experiments to a new video experiment with the same task and robot and to new experiments performed with a different type of robot (a mobile robot) in both video and live experiments. Thus, we were able to study the effect of politeness on user’s perceptions (enjoyment, trust, satisfaction) while considering users’ age group, type of robot or task, and study condition (live or video) and ensure results are not context-specific.

The next section discusses approaches to the study of politeness, emphasizing Lakoff’s approach to the concept and its relevance to non-humanoid robots. This section also reviews related work on the effects of age, type of robot/task and study condition in HRI studies, explaining the rationale for the current study. This is followed by methodology (Sect. 3), analysis and results (Sect. 4), discussion (Sects. 5 and 6), and conclusion (Sect. 6) sections.

## Theoretical Background and Related Work

### Politeness

The prominent studies of human politeness had been studied from a traditional point of view in sociolinguistics or pragmatic literatures. The seminal [3, 22–24] and the following works highlights politeness in a conceptual way. These studies use analytical tools implemented in observational examples. Most previous research on politeness in HRI has adopted Brown and Levinson’s [23] theory of politeness, which centers on the concept of “face.” The gist of the theory is the protection of ‘face’ or image by the social actors in a public domain. According to this theory [23], there are four strategies that a person could take to mitigate the “face-threatening” acts. The person can mitigate the situation by using an on or off-record strategy (on-record includes bald, positive and negative strategies; off-record strategy is to be indirect, using irony or metaphor). An actor can go on-record either without a redressive action (actions which are taken to minimize or overcome the intention of face-threatening) termed as bald strategy (being direct and clear in its strategy) or with a redressive action which includes two different strategies, namely positive and negative. In positive strategy, the face-threatening act is minimized by agreeing, being friendly, being optimistic etc. whereas in negative strategy, the face-threatening act is minimized by avoiding conflicts in showing consideration. Based on this theory, a humanoid mobile robot was used to remind a user about medication while the user was busy with a primary task [17]. The study involved four types of polite strategies i.e., bald, positive, negative, and a mixed strategy (combination of positive and negative). Results revealed that negative and a mixture of positive and negative strategies were recommended for polite behaviors. The positive strategy in Brown-Levinson theory was discouraged. A series of studies, which includes showing static pictures and animated clips to the participants, with a gatekeeper (peacekeeper) robot interacting with a human revealed that a polite strategy influenced the interaction [16]. The participants noted that the robot with the polite behavior was friendlier, fairer, and acted appropriately. It also revealed that the polite robot was less threatening irrespective of the static picture and animated clip. Another robotic receptionist study [13, 15] incorporated Brown and Levinson’s theory to develop a polite strategy with positive politeness. The study applied the bald strategy for the control group in two tasks: a chitchat task and a direction giving task. The implemented polite behavior did not affect the HRI performance in the direction giving task. However, in the chitchat task as well as in the direction giving task the polite behavior with a humanoid robot impacted positively the user perception. A study on compliance with a robot in relationship to speech and gesture features that express politeness suggested the need to develop multimodal levels of politeness since too much politeness caused negative impact [14]. The polite gestures, however, were positively associated with the social robot’s compliance. In a study in which adaptive feedback was implemented for a companion robot [18] the polite strategy was favored by the male participants while female participants preferred the direct commands. Another study examined the impact of impolite behavior on the performance of the participants in a physical trainer exercise [25]. The researchers found that the impolite robot (which was actually implemented as a rude robot) was not preferred by the participants. However, it yielded improved performance probably since it challenged the users. These studies support the hypothesis that polite behavior is preferred while interacting with a robot. However, all studies used humanoid robots. It is therefore crucial to expand the evaluation of politeness to other types of robots.

The concept of “face” and its implications for politeness rules may not be particularly suitable for human–machine interactions for several reasons. First, the strong emotional content associated with the face concept appears too strong for human–robot relations. Second, politeness based on face-saving strategies relies on verbal communication, whereas much of the interactions between humans and robots rely on nonverbal actions. Third, the face concept is highly sensitive to cultural variations [19, 26]. Furthermore, it is inapplicable to many HRI tasks in which the robot does not include a face (such as industrial and other tasks). Finally, Brown and Levinson’s theory is relatively complex, and cannot be easily transformed into HRI design guidelines.

To circumvent some of these issues, Bar-Or et al. [20] proposed a theoretical framework for politeness in the field of HCI which was inspired by Lakoff’s theory of polite behavior [3]. In the context of HCI, they demonstrated that polite behavior has a positive impact on user perception and efficiency. However, it remains to be seen whether Lakoff’s work can be applied to the design of social robots and its effect on aspects of human–robot interaction.

Lakoff [3] suggests three rules for polite interaction: (1) *Don’t impose* your actions or views on other people (at least not without first asking for permission); (2) *Give options* to other people to let them make their own decisions; and (3) *Be friendly* while interacting with other people, in the sense of producing at a sense of equality between the parties. Compared to other prominent politeness theories [24, 27], we consider Lakoff’s theory better suited for HRI research because it covers not only nuances of verbal interactions but also more general behavioral communication, which is an important aspect of social robotics. Unlike previous work on politeness in HRI, in which interaction was with a humanoid robot, the current study focuses on developing and evaluating polite behaviors in collaborative tasks with non-humanoid robots—a robotic arm and a mobile robot. Further, we focus on politeness in the interaction itself (and not on polite robot behaviors related to motions and gestures such as approach distance, angle and speed).

To provide a comprehensive analysis of the influence of the robot’s polite behaviors, we investigated several parameters as detailed below. For this study, we conducted both live experiments and video-based experiments to isolate the effect of the moving robot and to focus on the interaction aspects. The current study also includes a diverse population to explore the impact of politeness among different age groups, namely old and young adults. Lastly, we included two tasks (and related robots) to test the impact of polite behavior irrespective of the robot or task.

### Study Condition: Remote Versus in Situ

The Covid-19 pandemic posed serious limitations on our ability to conduct ordinary HRI research. But as sometimes is the case, it also offered an opportunity to enrich the research scope and methods. Therefore, we conducted two types of experiments—one in video, during periods of strict social distancing, and one in situ, during periods of relaxation in social distancing measures. Beyond the practical constraints, the use of a remote (video) study was motivated by findings of a previous study [21], which pointed out that participants (old adults) were more focused on the robot actions rather than concentrating on the interaction medium. However, as mentioned above, our goal was to assess people’s perceptions of the interaction rather than the robot’s physical movements. A video experiment was supposed to mitigate the saliency of physical activity and to help users focus on interactivity. The general guideline for conducting the remote experiment during the COVID-19 pandemic time has been demonstrated in [28]. Previous studies [29, 30] suggested that video experiments could be used for exploratory studies in HRI. These studies evaluated the preferable approach direction for a robot in both video and live HRI trials and revealed comparable people’s perceptions. However, both studies were limited to a university population which might have influenced the results (in [29], 15 participants aged 21–56 with many of them with computer sciences or robotics background; in [30], 42 university students and staff, aged 18–56). These studies suggested that videos could be used for HRI exploratory studies. Simultaneously, however, they noted limitations—the more the interaction between the robot and the study participant in a trial the less suitable a video would be since it lacks important aspects of the interaction such as dynamics, embodiment, and contingency [30]. Thus, we conducted *experiments both with a video and a live experiment*. In the video experiment, the users interacted with a robot that was remote from them. In the live experiment, the users interacted with a robot that performed the task in front of them.

### Effect of Age and Gender on Interaction

Effectiveness of assistive robots highly depends upon the acceptance and adoption by the users [31]. Far from the common perception that old adults are wary of technology [32], it was found they are open to new robotic technologies [33, 34]. Attitudes regarding the robot, either regarding the social impact and comfort of the robot or negative towards the robot, were similar in case of old, middle-aged and younger adults [35]. The older old adults (75–84 years) found that a physical training robot (‘Gymmy’) was more useful as compared to their 65–74 year old counterparts [36]. As aforementioned, politeness has been explored with different age groups [16]. However, the results did not reveal any significant effect of participants’ age on the user perception of the robot.

Nevertheless, in the current work, we relate to the effect of participants’ age on their perception. In a person following feedback design study it was observed that perception, preferences and the attitude of users towards the robot highly depends on age and gender of the user [37]. A comparative study between a real and virtual humanoid robot [38] revealed that a greater number of old adults complied with the real robot and had positive impression of both robots but felt more attached to the virtual robot. A survey aimed to assess preferences of robot tasks among old and young adults found that old adults anticipate more benefits of monitoring-type robots [39]. Another study with old and young adults interacting with a humanoid robot in a cognitive training task revealed that the design of the robot and interaction should be adapted to the user’s age and needs [40]. A previous preliminary study [21] in which we implemented polite behaviors for a robot manipulator revealed that young adults were able to differentiate between politeness levels. However, the old population was not able to do so. Both populations, though, indicated a preference for the polite behaving robot. All the above, barring one [16], suggested age is a relevant factor of interaction in HRI.

Previous research pointed out that gender influences perceptions of robots’ behavior [16, 18, 36, 41–45]. These studies suggested that male and female participants perceive the interaction with the robots differently. On the one hand, it has been suggested that male users are more aware of technological advancements than female counterparts [44]. Hence, male users tend to adapt to usage of robots more easily. This was also supported in [18], which was discussed in previous section, and included a comparison between male and female participants. The study found that the male participants perceived the polite robot more positively than female participants. On the other hand, [45] have found that female participants perceived the interaction with the robot more positively than male users, whereas the effect of gender on user’s perception of a polite gatekeeper was not significant [16]. Based on these ambiguous findings, we did not expect to find gender effect in our study.

### Type of Robot or Task

HRI taxonomy is classified into three categories: interaction context (e.g., field of application, type of interaction), robot (e.g., task of the robot, morphology) and team classification (e.g., role of each agent, composition of the team) [46]. In Sect. 2.2, we discussed the interaction context, specifically with an experiment in video and live conditions. In addition, this work involves *the usage of different types of robot* i.e., mobile and manipulator. Previous research mostly concentrated on comparing different anthropomorphic type robots [46, 47]. However, since the type of robot and task influences the interaction [49] it is important to consider this in the evaluation. Hence, we evaluated the effect of politeness levels in two different robot tasks/types.

The task of both robots was to bring the utensils for table setting. However, there was a difference in the task type performed by the robot. In one case, the robot manipulates the utensils in the environment to achieve its goal (using the manipulator the robot brings the utensils in front of the user). In the other case, the robot transports the utensils from one place to another (the mobile robot transports the utensils from one room to another where the user is sitting).

### Research Questions

The present study aims to investigate the influence of polite robot behaviors on HRI evaluations when using different types of robots (stationary and mobile), in varying conditions (video and live), and in different age groups (old adults and young adults). The following questions were investigated in all four studies:

1. Are participants able to perceive the differences in polite levels (irrespective of robot type, study condition, and age and gender)?
2. Do participants prefer the polite behavior of the robot (irrespective of robot type, study condition, and age/gender)?

Though previous research revealed that differences in some measures depend on the type of robot or task, the overall level of automation had an effect on the interactions irrespective of the robot or task [49]. Further research showed a similar impact of video and live experiments on how users perceived the interactions with the robot [25, 26]. Additionally, previous research [16] points out that the polite behavior of the robot was preferred irrespective of the participants’ age. Using Lakoff’s politeness rules as a blueprint for the design of interactive robots, we believe that participants would be able to find the difference between the three different polite behaviors and that they will prefer the politest behavior irrespective of their age, type of robot, and experimental condition.

## Methodology

### Model and Design

Based on Lakoff’s theory of politeness (see Sect. 2.1 above), three different levels of robot politeness in human–robot interaction were developed for a table setting task and implemented with two different types of robots (a robotic manipulator and a mobile robot) as follows:

Three-rules politeness level—all three rules (“*don’t impose*,” “*give options*,” “*be friendly*”) were applied.

Single-rule politeness level—only the “*don’t impose*” rule was applied.

No-rules politeness level—none of the framework’s rules was applied, although the robot did not explicitly or ostentatiously violate them.

### Experimental Setup

Both robots were programmed in Python and executed on ROS (robot operating system). In both experimental setups, the three different politeness levels were represented by three different colored buttons on a specially designed GUI. The experimental setup was designed to simulate a real-world task. This experimental design is similar to previous HRI studies [37, 50, 51]. A green colored button represented “no-rules” politeness level, a blue colored button represented “single-rule” politeness level, and a red colored button represented “three-rules” politeness level. The participants were not aware which color represented which level. Each participant went through all politeness levels: In the video experiment, the options were pre-selected in random order. However, in the live experiment, the user had to choose each option. Three videos of each trial (representing three levels of politeness for each of the two robots) were recorded for the video experiment. All the experiments were approved by the university ethical review process.

#### Robot Manipulator

**Fig. 1:**
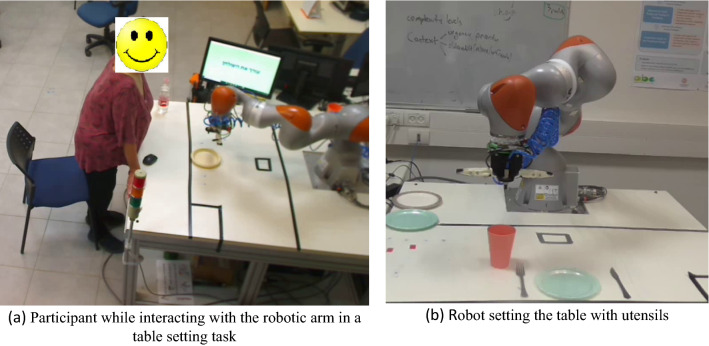
Manipulator robot experiment

A table setting task was designed with a KUKA LBR IIWA 14 R820 (7 degrees of freedom) manipulator. A TCP/IP protocol was developed for communication between the GUI and the robot. The participant had to interact with the GUI as shown in Fig. 1a. The robot offered two options for setting the meal—either a meat meal or a dairy meal.^1^ In each setting, it would bring a plate (either dairy or meat plate), a cup, a fork and a knife, as shown in Fig. 1b. The suction and the gripper were mounted on the robot to pick up the utensils. The three different polite behaviors are described in [21] and in Table 1.

**Table 1:** The GUI of the screen communicating with the user in manipulator robot experiment

Politeness level / Function	No-rules politeness level	Single-rule politeness level	Three-rules politeness level
Start	The robot starts setting up the table Display: Setting the table	Display: Do you want to set the table now? Option 1: Yes Option 2: No If option 1 is selected—the next window appears (Type of meal) If option 2 is selected—a time counter of 60 s is activated. At the end the same window re appears	Display: Hello I am robot KUKA. I would be happy to set the table for you Option: Next New window appears after selecting Next Display: When do you want to set the table? Option 1: Now Option 2: In a minute Option 3: In two minutes If option 1 is selected—the next window appears A time counter is activated for options 2 and 3 according to selection and then the window re appears
Type of meal	Dairy	Display: Do you want the robot to set meat meal utensils? Option 1: yes Option 2: No If option 1 is selected—the robot brings meat meal utensils If option 2 is selected- the robot brings dairy meal utensils Display: Setting the table	Display: Would be happy to know what kind of meal you prefer to set the table for? Option 1: Meat Option 2: Dairy Option 3: I don’t care The robot brings respective utensils based on options 1 and 2. For option 3 it brings dairy utensils Display: Setting the Table
Stop	Display on screen: Finish	Display: Are you satisfied with the set table? Option 1: Yes Option 2: No If option 2 is selected—the robot exchanges plates Display: Finish	Display: I am finished with setting the table. Are you satisfied with arrangement? Option 1: Yes (with thumbs up emoji) Option 2: No, I would rather change the plates (with thumbs down emoji) If option 2 is selected—the robot exchanges plates Display: Thank you very much. Bon appetite

#### Mobile Robot

**Fig. 2:**
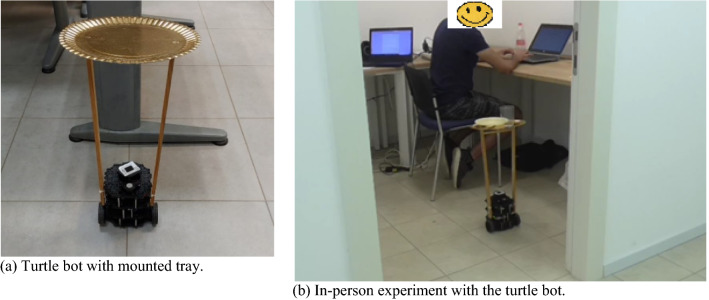
Mobile Robot Experiment

A small mobile robot (Turtlebot3 Burger with two motorized wheels and a LIDAR used for navigation) was used to bring dishes and cutlery for the table setting task. To bring the utensils, a wooden structure with a tray on top of it was mounted on the robot, as shown in Fig. 2a and b. The height of the wooden frame was 42 cm, and the diameter of the tray was 29 cm. The interface was developed in HTML and JavaScript. The dialogue communicated by the robot to the user is described in Table 2. The difference in this experiment was that the robot asks about the number of utensils required to set the table (according to the number of people dining) instead of asking about different types of meal and the time required to set the table (as in the case of the manipulator robot experiment). In the case of manipulator robot, the participant could easily differentiate between the types of meal as the utensils were present in front of them. However, in the mobile robot experiment the robot would move outside the experimental area to bring the utensils, hence the option of number of utensils was chosen for this experiment.

**Table 2:** The GUI of the screen communicating with the user in the mobile robot experiment

Politeness level/Function	No-rules politeness level	Single-rule politeness level	Three-rules politeness level
Start	The robot would go and bring the utensils Display: Bringing the utensils	Display: Please click start to bring up a set of utensils After pressing start the robot would bring the utensils Display: Bringing the utensils	Display: Hello! I'm a robot helping to serve the utensils to the table. Please click "Start" when you are ready Next window appears after choosing Start
Number of people	For one person	After bringing the utensil, there would be display on screen: Please click start to bring another set of utensils. Buttons: “Start” or “Finish” If Start is pressed the robot would bring another one, else would finish the task	Display: How many people do you want me to bring utensils for? Option 1: For one people Option 2: For two people After one of the above options is clicked the robot would bring the utensil depending on the option selected Display: Bringing the utensils
Finish	Display on screen: Finish	Display on screen: Finish	Display: Do you want me to bring another set? Option 1: Yes Option 2: No If option 1 is selected it would bring another set and then task is finished If option 2 is selected the task is finished Display on screen: “Thank you very much”

### Procedure

#### Video Experiments

We sent the video experiments’ participants a zoom link (a video conference environment) to connect with the experimenter. Once connected, the participants first gave their consent and then filled out a preliminary questionnaire (the consent instructions and questionnaire were prepared in Google Forms and the link to the form was shared with them). We explained the task and asked them to watch the videos. The videos were presented in a different order for each participant to maintain the random effect. After watching each video, we asked the participants to fill the post-trial questionnaire (see Appendix), and by the completion of the experiment they answered the final questionnaire (see Appendix).

#### Live Experiments

After arriving at the experiment area, the participants were asked to sign a consent form. Then they were asked to fill a preliminary background questionnaire. The participants were then briefed about the experiments with the robot. They were free to start with any color (on the aforementioned GUI) to maintain randomness and were not informed about the representation of each color. After clicking one of the colored buttons, the robot performed its task according to the chosen scenario. After completing the task in a particular scenario, participants were asked to fill the post-trial questionnaire (see Appendix). On completion of all the three scenarios, participants were asked to fill the final questionnaire (see Appendix).

### Participants

Overall, 203 people participated in the eight experiments, of whom 97 participated in the manipulator experiments and 106 in the mobile robot experiments. The age and gender of all participants are detailed in Table 3. Old adults were recruited through online advertisement in social media, snowball sampling and personal telephones to participants who participated in previous experiments in our lab. Young adults were recruited through online advertisement in a mandatory academic course; participation was voluntary, and every participant received compensation in the form of a bonus point contributing to a credit in the academic course. All young adults had experience with both computers and robots. The young adults who participated in the live experiment were invited to the university. In the case of old adults, the experimenter visited the participants' homes in accordance with the restrictions during the COVID-19 pandemic. The video experiment was conducted via zoom for both populations because of the limited access to the participants due to lockdowns during the COVID-19 pandemic. The ensuing statistical analyses adjusted for the disparity in sample sizes, which was mainly due to the restrictions imposed during the Covid-19 pandemic.

**Table 3:** Participants age and gender in the experiments

Experiment	Robot Type	Sample	Type	Sample size	Age (avg ± std)	males	females
1	Manipulator	Young adults	Live	30	25.87 ± 5.62	11	19
2			Video	27	25.67 ± 1.84	12	15
3		Old adults	Live	20	73.85 ± 4.99	8	12
4			Video	20	75.31 ± 4.41	10	10
5	Mobile robot	Young adults	Live	22	26.45 ± 2.53	11	11
6			Video	44	25.45 ± 1.61	22	22
7		Old adults	Live	20	69.38 ± 3.20	7	13
8			Video	20	70.20 ± 3.75	10	10

## Results

The post-trial questionnaire included three subjective measures: enjoyment, satisfaction, and trust. The items in the questionnaire were selected to evaluate the perception of the users while interacting with different levels of robot politeness. Thus, the items in the questionnaires are general in the sense that they measure perceptions of politeness, they do not focus on the sources of politeness. The sources of politeness are different in terms of actual behaviors of two different robots in two different settings. However, those behaviors can be abstracted to be described by the three rules of Lakoff’s model. Hence, based on this abstraction, we can conclude and generalize about the contribution of those rules to people’s perceptions of robot politeness. The items related to the measures are detailed in the Appendix. The average for each measure was computed if there was more than one item related to the measure. The questionnaires were intentionally kept short to avoid participants’ fatigue along the experiment (there were three interacting sessions with the robot for each participant). The items were selected from various sources including the HCI domain, e-commerce, autonomous vehicles and assistive robotics and were adapted to the current study.

The items for enjoyment were adapted from [20, 52–54]. The items for satisfaction were adapted from [20, 55]. The items for trust were adaptations from [56] on autonomous vehicles and [57] on assistive social agents. The items in post-trial questionnaire A were used only for the live experiment with the manipulator robot, which included two groups (see Table 3) and were conducted before COVID-19 pandemic. While reorganizing our research plan amidst the pandemic outbreak, we found that there was a need to shorten the questionnaire and revise some of the questions. This resulted in post-trial questionnaire B that was used for the rest of the experiment. Post questionnaire B includes two items of enjoyment (between item correlation = 0.69), one item of satisfaction and two items of trust (between item correlation = 0.82). The fact that questionnaires were not identical is a limitation. However, the results from the first two studies are consistent with the rest of the studies, suggesting that the dependent variables' essence was captured similarly by both questionnaires.

Descriptive statistics (mean and standard deviation) were computed for each of the dependent variables. Further, the two-sample Kolmogorov–Smirnov test was conducted on each dependent variable to check for normal distribution. Results revealed that all three dependent variables i.e., enjoyment (D = 0.15, *p* < 0.001), satisfaction (D = 0.18, *p* < 0.001) and trust (D = 0.20, *p* < 0.001) were not normally distributed. Hence, we conducted ordinal regression with a cumulative link mixed model. To compute ordinal regression, the response evaluated from the dependent variables were rounded to the nearest integer resulting in 5 ordinal levels (strongly agree, agree, neutral, disagree and strongly disagree).

The independent variables were defined as participants’ age, study condition, type of robot and level of polite behavior. The model was fitted with stepwise elimination using the “buildmer” [58] package in R studio. The scores received from the participants were labeled according to all the independent variables. The regression analysis was adjusted for the different sample sizes of the various experimental groups. Post-hoc test was conducted using least square means with Bonferroni correction. The level of significance (α) was set to 0.0167 after Bonferroni correction.

In addition, to evaluate whether polite behavior is preferred, a logistic regression was computed in which the dependent variable was the preference for the polite level, as expressed in the final questionnaire. The three different levels of politeness were divided to (1) polite behavior (“three-rules” politeness level) and (0) “ordinary” behavior (“single-rule” and “no-rules” politeness levels). The participants’ age, type of experiment and type of robot were the independent variables. The model was fitted in stepwise elimination using the “buildmer” package [58].

The results are presented for each dependent variable, namely enjoyment (Sect. 4.1), satisfaction (Sect. 4.2), and trust (Sect. 4.3). This section also includes the discussion on preference (Sect. 4.4) among the three levels based on age, type of experiment, and study condition. Results revealed that gender did not have a significant effect on any of the dependent variables (Enjoyment z-ratio = 1.634, *p* = 0.1022; Satisfaction z-ratio = 1.417, *p* = 0.1564; Trust z-ratio = 1.792, *p* = 0.0731 and Preference z-ratio = −1.113, *p* = 0.2657). Consequently, we do not elaborate on this variable in the subsequent analyses.

A detailed analysis conducted for each independent variable, namely different levels of politeness, participants’ age, study condition, and type of robot/task is presented in the discussion section.

### Enjoyment

**Fig. 3:**
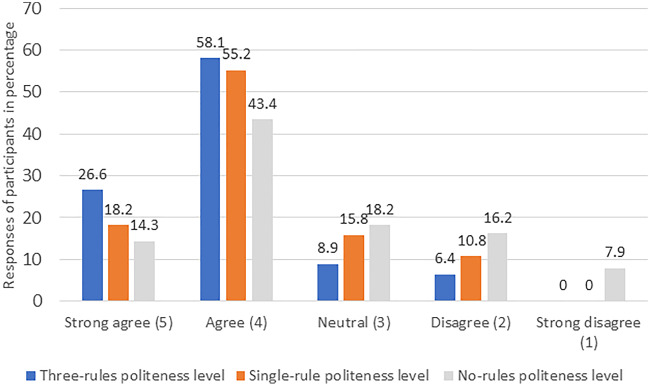
The participants’ responses (%) to the enjoyment items for different politeness levels

Most participants enjoyed the interaction when the “three-rules” politeness level was employed, as shown in Fig. 3 (the percentage of people agreeing or disagreeing with the questions after averaging and rounding it to nearest integer related to enjoyment). Less participants enjoyed the interaction with the “single-rule” level, as shown in Fig. 3. Further, in the “no-rules” politeness level the least number of participants enjoyed interacting with the robot as shown in Fig. 3. The mean and the standard deviation in case of enjoyment is reported in Fig. 6. More people in the live format (27.2% in strongly agree, 46.0% in agree, 16.7% in neutral, 8.3% in disagree, and 1.8% in the strongly disagree category) enjoyed the interaction with the robot as compared to the video format (13.5% in strongly agree, 57.4% in agree, 12.3% in neutral, 13.5% in disagree, and 3.3% in strongly disagree category) enjoyed the interaction with the robot. The significant explanatory variables for fitting the ordinal regression model were study condition (video vs live) (z = −2.43, *p* = 0.015), participant’s age (z = −2.15, *p* = 0.032) and levels of polite behavior (single-rule compared to no-rule: z = 5.33, p < 0.001 and three-rules compared to no-rule: z = 8.34, *p* < 0.001). However, the age of the participants was not significant (*p* > 0.017), hence it was eliminated from the model. The post hoc test for the within groups found that participants easily differentiated between the three different politeness levels (see Table 4).

**Table 4:** Post hoc analysis comparing the three polite levels for each dependent measure

Three levels of politeness	Enjoyment	Satisfaction	Trust
No-rules vs. “single-rule” politeness	z. ratio = −5.33, *p* < 0.001	z. ratio = −86.91, *p* < 0.001	z. ratio = −4.76, *p* < 0.001
No-rules vs. “three-rules” politeness	z. ratio = −8.34, *p* < 0.001	z. ratio = −127.33, *p* < 0.001	z. ratio = −5.78, *p* < 0.001
“single-rule” vs. “three-rules” politeness	z. ratio = −3.90, *p* < 0.001	z. ratio = −31.43, *p* < 0.001	z. ratio = −1.21, *p* = 0.447

### Satisfaction

**Fig. 4:**
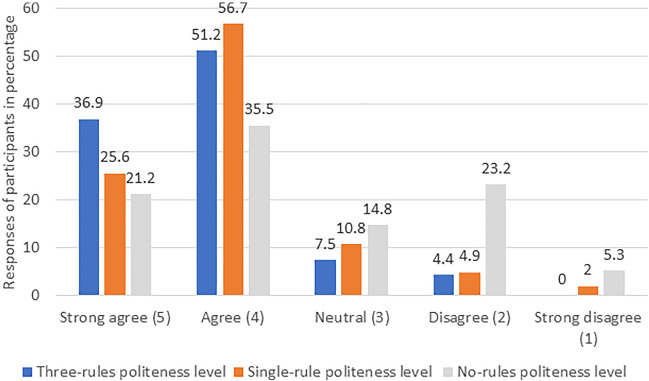
The participants’ responses (%) to the satisfaction item for different politeness levels

Most participants were satisfied while interacting with the robot at the “three-rules” politeness level, as shown in Fig. 4 (the percentage of people agreeing or disagreeing with question related to satisfaction). Less participants were satisfied while interacting with the robot employing “single rule” politeness level, as shown in Fig. 4. The least number of participants were satisfied interacting with the robot employing “no rule” politeness level, as shown in Fig. 4. The mean and standard deviations are reported in Fig. 6. More participants were satisfied while interacting with a mobile robot (32.4% in strongly agree, 54.4% in agree, 4.1% in neutral, 7.6% in disagree and 1.5% in strongly disagree category) as compared to the manipulator (23.0% in strongly agree, 40.6% in agree, 18.6% in neutral, 14.4% in disagree and 3.4% in strongly disagree category). The independent variables i.e., level of polite behavior (single- rule compared to no-rule: z = 86.97, *p* < 0.001 and three-rules compared to no-rule: z = 127.33, *p* < 0.001) and the type of robot (z = 86.82, *p* < 0.001) were best fitted for the ordinal regression model. The post-hoc test reflected the difference in all the three levels when pairwise comparison was taken, as reported in Table 4. The post hoc test revealed that participants easily differentiated between the three different politeness levels.

### Trust

**Fig. 5:**
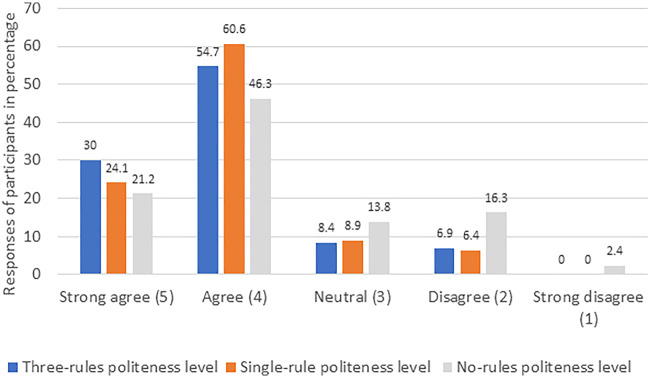
The participants’ responses (%) to the trust items for different politeness levels

**Fig. 6:**
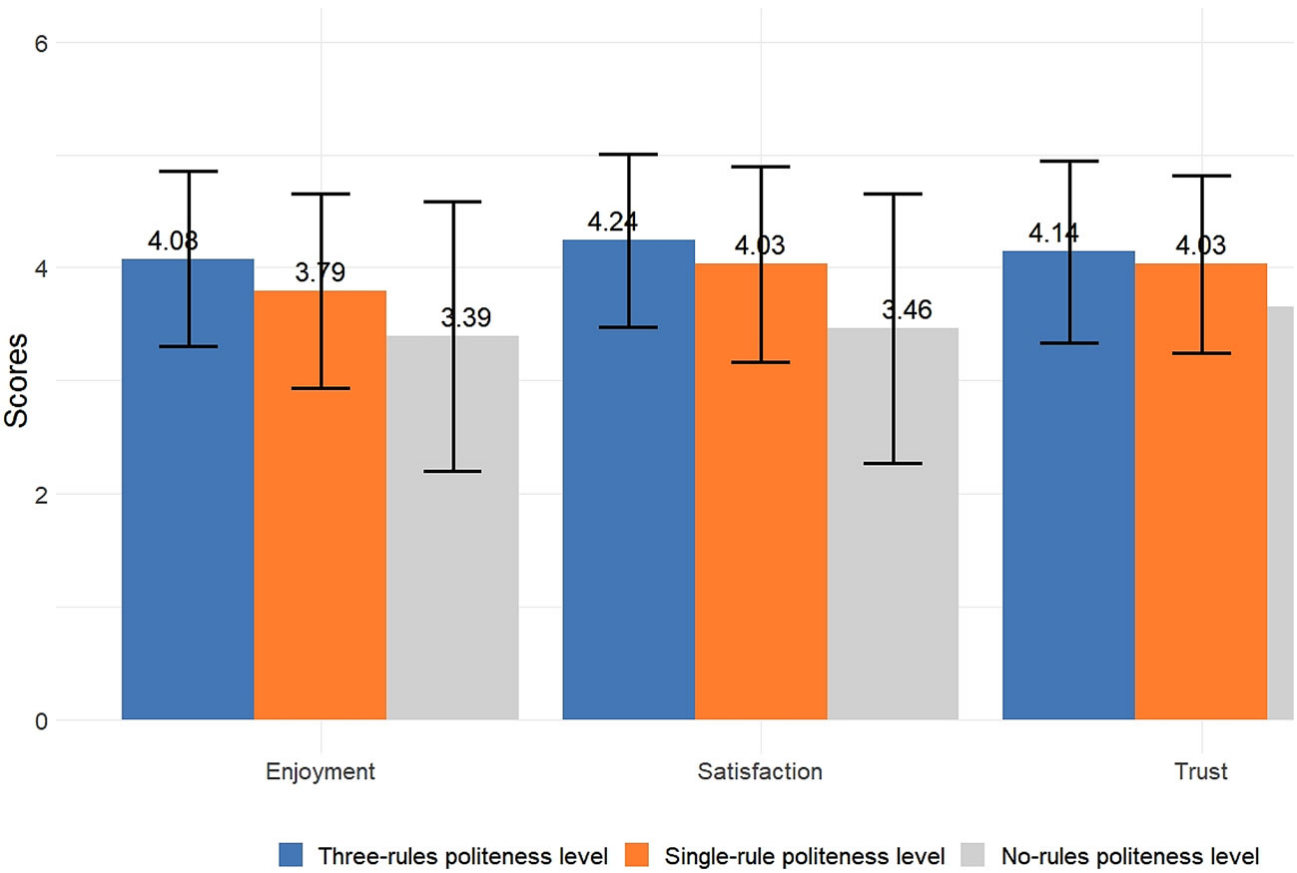
Mean and standard deviation (error bars) of the independent measures for the different politeness levels of the robots

It was found that most participants trusted the robot when the “three-rules” politeness level was employed by the robot, as shown in Fig. 5 (the percentage of people agreeing or disagreeing with questions after averaging and rounding to nearest integer related to trust). Most participants also trusted interacting with the robot when the “single-rule” politeness level was employed, as shown in Fig. 5. Least participants found the robot with “no rule” politeness level to be trustworthy, as shown in Fig. 5. The descriptive statistics for trust in all three different levels of polite behavior is represented in Fig. 6. It was observed that in the live experiment (30.8% in strongly agree, 55.1% in agree, 8.7% in neutral, 4.7% in disagree and 0.7% in strongly disagree category), participants found the robot more trustworthy compared to the video experiment (20.4% in strongly agree, 52.9% in agree, 11.7% in neutral, 14.1% in disagree and 0.9% in strongly disagree category). Old adults (34.2% in strongly agree, 48.8% in agreeing, 10.0% in neutral, 5.8% in disagree, and 1.2% in strongly disagree category) found the robot more trustworthy compared to young adults (19.2% in strongly agree, 57.2% in agree, 10.6% in neutral, 12.5% in disagree and 0.5% in strongly disagree category). The regression analysis revealed that polite behavior levels (single-rule compared to no-rule: z = 4.76, *p* < 0.001 and three-rules compared to no-rule: z = 5.67, *p* < 0.001), study condition (z = −2.82, *p* = 0.0048) and age of participants (z = −2.56, *p* = 0.01) were the best fitted model. The post-hoc test for trust within three independent variables appears in Table 4. The results indicate that participants were able to differentiate in most pairwise comparisons except for “single-rule” and “three-rules” politeness levels.

### Preference

In the final questionnaire, 74.9% of the respondents reported that they noticed the difference between the politeness levels. It is evident from Figs. 3, 4, 5 that the three-rules politeness level was preferred among the three levels for all three dependent variables. Descriptive analysis of the final questionnaire revealed that 76.4% of the young adults and 48.8% of the old adults preferred the robot behavior in the “three-rules” politeness condition, 13.8% of the young adults and 23.8% of the old adults preferred the robot behavior in the “single-rule” condition, whereas 9.8% of the young adults and 27.4% of the old adults preferred the “no-rules” politeness condition. Logistic regression revealed that age had a significant effect on preference (z = 3. 98, *p* < 0.001). In addition, the following was the participants’ response when asked about the *least* preferred condition (Item 3 of the final questionnaire): 64.5% selected the “no-rules” politeness level, 16.8% selected the “single-rule” politeness level and 18.7% selected the “three-rules” politeness level.

## Discussion

To the best of our knowledge, this study is the first to adopt Lakoff’s approach to politeness [3] in the domain of social robotics and HRI. The main potential advantages of this approach are its relative simplicity (being based on three fundamental rules) and its applicability to non-verbal interactions. This is especially important in interaction with non-humanoid robots. The results indicate that manipulating politeness according to this approach was successful. Importantly, we tested the generalizability of the findings by using two different robots and two experimental modes—live and by video. Finally, we also tested the effects of age and gender. The design of the experiment which included three levels of polite behavior, two types of study conditions and two types of robotic tasks might seem a bit complex. However, the user only interacted with one robot and three levels of polite behavior at a time.

The results revealed that robots applying all three politeness rules were rated higher than the other levels on all three dependent variables (enjoyment, satisfaction, and trust). Further, the participants enjoyed the interaction in the live format and found it more trustworthy than the video format. In addition, the participants were more satisfied while interacting with the mobile robot as compared to the manipulator. We also found that old participants had more trust in the robot than the young participants. Contrastingly, more young participants preferred the most polite (the three-rules) robots than old participants. In this section, we discuss the implications of the results in terms of the effects that the independent variables had on users’ perceptions and preferences.

### Polite Behavior

We developed three levels of polite behavior for assistive, non-humanoid robots, based on Lakoff’s (1973) theory. Results from the study demonstrated that participants were able to perceive the difference among the three different politeness levels in terms of enjoyment, satisfaction and trust. They also preferred the polite behaving robots. It is evident from the results that most participants preferred the robots that followed the “three-rules” condition, although they could not explicitly differentiate between “single-rule” and “three-rules” politeness levels in the case of the trust measure. The result in [9, 11], which employed Brown-Levinson’s politeness theory [23], showed that polite behavior was not effective in improving HRI performance. Our study demonstrates that participants were satisfied, enjoyed the interaction, and trusted the robot that employed polite behavior. This corresponds to the gatekeeper robot study [16], where most participants preferred the polite behavior condition. However, the gatekeeper robot study lacked interactions with a real robot (it was based on animated interactions to judge the robot’s politeness). Further, both aforementioned studies [13, 15] were based on verbal interaction with a robot. However, much of the interaction with the robot is based on its action. A noticeable advantage of using Lakoff’s rules is that, as demonstrated in the scenarios used in our study, they are relevant to the non-verbal behavior of assistive robots.

### Study Condition: Remote Versus in Situ

The regression analysis (Sects. 4.1 and 4.3) revealed that study condition (remote, video vs in-situ, live) influences enjoyment and trust in HRI. Further, the descriptive analysis (Sect. 4.1 and 4.3) revealed that both in the case of enjoyment and trust measures, participants perceived the live type of experiment more suitable as compared to the video experiment. Previous works [29, 30] suggested that video experiments have a similar effect on HRI performance as live experiments. However, the researchers cautioned that video experiments would not be able to replace the live experiments in a more interactive environment. In our work, the enjoyment and trust levels when interacting with a live robot were higher than in the video experiment. Nevertheless, both conditions were useful in revealing the impact of politeness on users’ evaluations. Therefore, it seems that both video and live experiments can be used to analyze the impact of social traits in HRI due to their advantages. However, findings from video studies may indicate somewhat weaker effects.

### Effect of Age and Gender on Interaction

The regression analysis (Sects. 4.3 and 4.4) revealed that the participant’s age was a factor in fitting the model of trust in the robot and preference its behavior. The descriptive analysis showed that old adults perceived the robot as more trustworthy compared to young adults. This could possibly be due to their lack of knowledge of possible limitations and failures of robotic technology. Contrary to popular belief of old adults being skeptical of new technologies [32], research has shown that they are more open towards accepting new technologies [33, 34]. The young adults preferred the “three-rules” level over every other politeness levels. As pointed out in [40] the robot’s design should be based on participants age and their needs. Though in this study we employed the same design for both age groups. Similarly in [16] the design for all age groups was the same, the participants judged the interaction between an animated gatekeeper robot and an animated person recorded in a clip. However, participants were not directly involved in the interaction with the robot. Hence the age of the participants did not have influence over the user perception. With direct interaction with the robot, the design should be more influential; hence based on [40], we recommend the design to be age specific. In line with [16], in this study participants’ gender was not found as an influencing factor.

### Type of Robot or Task

The regression analysis (Sect. 4.2) revealed that the type of robot and task influenced satisfaction. The descriptive analysis indicates that more participants were satisfied with the mobile robot than the manipulator robot. This is in line with [49], who found that user perception was influenced by the type of robot or task. In the current study, this influence can be explained by the fact that the mobile robot brought all the utensils to the user in one attempt, whereas the manipulator robot brought the utensils one at a time. Since initially the utensils in the manipulator robot task were on the table, easily visible and within reach, the participants may have perceived the robot as slow. Participants complained about the robot’s speed in the manipulator robot experiment. Therefore, it is important when evaluating perception about the behaviors of a robot to consider different types of tasks and robots.

## Conclusions

A series of eight studies examined the consequences of designing polite behavior in non-humanoid robots. Previous HRI literature pointed out that anthropomorphic design of the robot increases the trust of the people in it. However, design complexity increases when incorporating humanoid systems. Further, many robotic applications employ non-humanoid robots. Hence, this study investigated politeness in non-humanoid robots. In order to ensure results are not valid for specific conditions we conducted a wide study including different robots, populations and conditions. The generalizability of the results can only be increased if similar effects are shown for different robotic systems (mobile robot vs. manipulator), scenarios (live and video) and populations.

The design of the robots’ behavior was based on the politeness rules introduced by Lakoff [3]. We suggested that these rules could be more easily translated into behavioral guidelines than politeness rules offered by other theories. Our original intent was to assess whether applying those rules of Lakoff to social robots is perceived accordingly and similarly by both young and older people. At an early stage of this research project, our work was disrupted by the outbreak of the Covid-19 virus and the ensuing of social distancing policies and practices. While the disruption hampered and delayed our planned standard in-situ experiments, it presented an opportunity to replicate the same planned studies using video clips and zoom sessions. Thus, we conducted the research in two conditions, video-based studies during times of social distancing and live experiments during periods when restrictions were relaxed. Consequently, we could also examine commonalities and differences in findings in the two conditions.

The main contribution of this work is the finding that people can distinguish degrees of politeness in the behavior of non-humanoid social robots designed based on Lakoff’s politeness rules. Hitherto research has mainly shown that people perceive politeness in verbal utterances or explicit gestures of humanoid robots. Here, however, we demonstrated the usefulness of our approach in eliciting a sense of politeness merely by designing the non-humanoid robot’s mundane interactive behavior.

The issue of age is highly relevant to research on social robotics because we expect different age populations to use social robots for different purposes and because we foresee older adults to be more frequent users of social robots compared to their use of other computing technologies. In general, the results suggest that both older and younger adults perceive politeness differences in the behavior of social robots, although there were some mild differences in proportions. Furthermore, both populations preferred robots that were designed with the three politeness rules over robots that were not designed with any of the politeness rules. Thus, our second contribution is that both older and younger adults value the polite behavior of social robots.

The third contribution relates to the comparison between the in-presence vs. video-based settings of the studies. Similar to previous studies, we found that the participants enjoyed the robots and trusted them more in the live condition. However, we also found that participants in both conditions were sensitive to the politeness manipulations and preferred the more polite versions of the robot. Thus, we suggest that future research on politeness may be less susceptible to the effects of video-based experiments relative to other aspects of HRI research.

We see this rigorous evaluation as a major contribution of this paper; it is important to evaluate HRI studies in a multitude of conditions and with different populations to generalize the results (vs. snapshot studies with limited population and single task—results might be too specific and tailored to the experimental conditions).

## Data Availability

The data set generated during and/or analysed during the current study are available from the corresponding author on reasonable request.

## Footnotes

1 There are different types of utensils for meat and dairy meal according to the common religious practice in Judaism.
